# A combination of LongSAGE with Solexa sequencing is well suited to explore the depth and the complexity of transcriptome

**DOI:** 10.1186/1471-2164-9-418

**Published:** 2008-09-16

**Authors:** Lucie Hanriot, Céline Keime, Nadine Gay, Claudine Faure, Carole Dossat, Patrick Wincker, Céline Scoté-Blachon, Christelle Peyron, Olivier Gandrillon

**Affiliations:** 1 UMR5167 CNRS Université Claude Bernard Lyon1, Université de Lyon, Institut Fédératif des Neurosciences de Lyon, 7 rue Guillaume Paradin, 69372 Lyon cedex 08, France; 2 PRABI, Université Claude Bernard Lyon 1, Bâtiment Gregor Mendel, 16 rue Raphaël Dubois, 69622 Villeurbanne Cedex, France; 3 UMR5534 CNRS Université Claude Bernard Lyon1, Université de Lyon, Bâtiment Gregor Mendel, 16 rue Raphaël Dubois, 69622 Villeurbanne Cedex, France; 4 Genoscope (CEA), 2 rue Gaston Crémieux CP5706, 91057 Evry, France; 5 CNRS, UMR 8030, 2 rue Gaston Crémieux CP5706, 91057 Evry, France; 6 Université d'Evry, 91057 Evry, France

## Abstract

**Background:**

"Open" transcriptome analysis methods allow to study gene expression without *a priori *knowledge of the transcript sequences. As of now, SAGE (Serial Analysis of Gene Expression), LongSAGE and MPSS (Massively Parallel Signature Sequencing) are the mostly used methods for "open" transcriptome analysis. Both LongSAGE and MPSS rely on the isolation of 21 pb tag sequences from each transcript. In contrast to LongSAGE, the high throughput sequencing method used in MPSS enables the rapid sequencing of very large libraries containing several millions of tags, allowing deep transcriptome analysis. However, a bias in the complexity of the transcriptome representation obtained by MPSS was recently uncovered.

**Results:**

In order to make a deep analysis of mouse hypothalamus transcriptome avoiding the limitation introduced by MPSS, we combined LongSAGE with the Solexa sequencing technology and obtained a library of more than 11 millions of tags. We then compared it to a LongSAGE library of mouse hypothalamus sequenced with the Sanger method.

**Conclusion:**

We found that Solexa sequencing technology combined with LongSAGE is perfectly suited for deep transcriptome analysis. In contrast to MPSS, it gives a complex representation of transcriptome as reliable as a LongSAGE library sequenced by the Sanger method.

## Background

Methods for transcriptome analysis are today diversified and can be divided in two families of technologies: "closed" and "open" techniques [1]. In closed technologies such as microarrays, the space of inquiry is finite since the analysis of the expression level is limited to previously characterized transcript sequences for which a corresponding probe was spotted on the microarray. In contrary, open technologies analyze the transcriptome without any *a priori *knowledge on the transcript sequences. These methods thus allow the discovery of new transcribed sequences [2-5]. This is particularly interesting as all transcribed sequences have not been discovered yet, even in well studied species like mouse [5] and human [3].

The most widely used methods for open transcriptome analysis are based on the sequencing of either cDNAs (known as Expressed Sequence Tags or ESTs) or of short tag sequences. This later strategy has been developed in Serial Analysis of Gene Expression (SAGE) [6], LongSAGE [4] and Massively Parallel Signature Sequencing (MPSS) [7]. They are by construction much more efficient in sampling the depth of the transcriptome than the EST sequencing techniques. In contrast to the 14 bp SAGE tags generated by SAGE, the 21 bp tags obtained either by LongSAGE or MPSS can directly be mapped to the genome sequence, which is particularly interesting for the identification of new transcribed sequences [4].

Today, the new challenge of gene expression analysis is the deep analysis of transcriptomes in order to investigate the role of weakly expressed genes that can nevertheless play an important role in different biological processes. A recent study revealed that millions of transcript tags have to be sequenced in order to fully characterize a human transcriptome ([8-10]). In this respect, the MPSS technique is particularly interesting as its bead-based sequencing technology allows to sequence simultaneously more than one million of tags in a library. This is therefore far more efficient and faster than the sequencing of a LongSAGE library by the Sanger method. However, recent publication shows that MPSS libraries are significantly less complex than much smaller LongSAGE libraries, revealing a serious bias in the generation of MPSS data ([11,12]).

**Figure 1:**
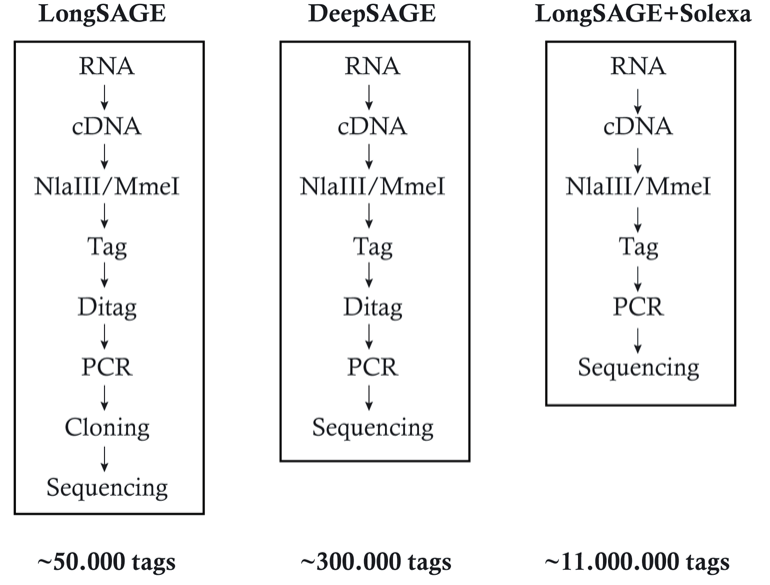
**Schematic illustration of the LongSAGE-Solexa procedure**. From left to right is shown the initial SAGE procedure ([6]), the improvements brought by the DeepSAGE procedure ([14]) in terms of a simpler protocol and of depth of sampling. On the right is reported the LongSAGE-Solexa procedure described in this study, which provided a major improvement along those two lines. One has to note that in the LongSAGE-Solexa procedure, a single tag is sequenced for each sequenced molecule.

It is therefore of great interest to design a new method combining a tag-based technique such as LongSAGE with a high throughput sequencing technology in order to perform deep transcriptome analysis and explore the large complexity of the transcriptome. A first combination of SAGE and 454 sequencing, called DeepSAGE, has been published ([13,14]) and leads to both increased sensitivity and less tedious library preparation (Figure 1). However, although DeepSAGE allows the counting of more than 300,000 tags, it is still based on creation of ditags [14]. We propose that the cost-effectiveness Solexa sequencing technology [15], which allows to sequence millions of short cDNA of 35 bp per sample, would lead to another major advance by still reducing the library construction time and increasing the sensitivity (Figure 1).

We therefore built up a library of male adult mice hypothalamus, a brain region involved in behavioral and autonomic coordination, using LongSAGE and Solexa sequencing technologies. In order to assess if we can properly explore the transcriptome complexity with this method, we compared this library with a LongSAGE library of mouse hypothalamus sequenced with the Sanger method. We found that, for the same number of tags, a comparable and even slightly higher level of complexity of transcriptome is uncovered with LongSAGE combined with the Solexa technology than with the Sanger method. Therefore, the combination of LongSAGE and Solexa sequencing seems to be perfectly suited for deep transcriptome analysis.

## Results and discussion

### Major characteristics of the two libraries

**Figure 2:**
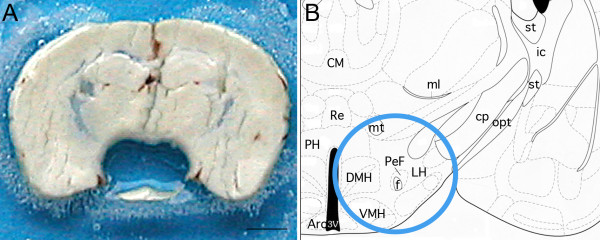
**Illustration of the extent of tissue collection**. A: Photograph of a frontal 400 μm-thick section of a mouse brain at the level of the hypothalamus. The hypothalamus, centered on the perifornical nucleus was collected bilaterally using a trocard of 1 mm diameter. Scale bar = 1 mm. B: Schematic drawing of the section presented in A and extracted from the mouse atlas of G Paxinos & KB Franklin, (+1,98 mm interaural). The blue circle highlights the extent of the brain area taken off. 3V: third ventricule; Arc: arcuate nucleus; CM: centro-medial thalamic nucleus; cp: cerebral pedoncule; DMH: dorsomedial hypothalamic nucleus; f: fornix; ic: internal capsule; LH: lateral hypothalamic area, ml: median lemniscus; mt: mammillothalamic tract; opt: optic tract; PeF: perifornical nucleus; PH: posterior hypothalamic area; Re: thalamic reuniens nucleus; st: stria terminalis; VMH: ventromedial hypothalamic nucleus.

Two libraries were created from male adult mice hypothalamus, collected bilaterally as punches centered on the perifornical nucleus from the caudal part of the paraventricular nucleus to the mammillary bodies (Figure 2). A first library was constructed by using the LongSAGE method combined with the Sanger sequencing technology (Sanger_Hypo), and a second library with the LongSAGE method combined with the Solexa sequencing technology (Solexa_Hypo) (Figure 1). Major characteristics of both libraries are summarized in Table 1.

**Table 1:** Major characteristics of the Sanger_Hypo and Solexa_Hypo LongSAGE libraries

	Sanger_Hypo	Solexa_Hypo
RNA origin	Hypothalamus	Hypothalamus
Mouse strain	Fvb	C57BL/6
RNA amplification	No	Yes
Sequencing method	Sanger	Solexa
Tag length	21 bp	21 bp
Total number of tags	68,023	11,017,712
Number of unique tags	23,007	609,407
Number of tags with only one occurrence	15,612	193,917
% of tags matching to the genome*	90.28	92.25

* We consider that a tag matches to the genome if it has 100% identity over its whole length (21 bp).

The Solexa_Hypo library is 162 fold deeper (11,017,712 tags) than the Sanger_Hypo library (68,023 tags). Far more unique tags were therefore obtained in the Solexa_Hypo library (609,407) than in the Sanger_Hypo one (23,007) (Table 1). With both the Sanger and the Solexa techniques, a very high percent of the sequenced tags matched to the mouse genome (90.28 and 92.25% of the Sanger_Hypo and Solexa_Hypo tags respectively, Table 1). This proportion is higher than a previous estimation (82.7%) computed by estimating the proportion of erroneous tags in LongSAGE libraries [16]. This could be due to our consideration of the base-call quality during the extraction of tags from concatemer sequences (see Method section). Nevertheless, it is to keep in mind that the proportion of tags that do not match to the genome is an over-estimation of the proportion of the erroneous tags. Indeed, several tags without errors do not match to the genome because they overlap two exons, extend into the polyA tail or contain polymorphic positions [3]. Furthermore, the probability that an erroneous tag match to the genome has been estimated to be very low [3]. Consequently, the tag sequences obtained in both Solexa_Hypo and Sanger_Hypo libraries seem to be of excellent quality, and the overall quality of the Solexa_Hypo tags seems to be slightly better than the Sanger_Hypo ones.

One could argue that the better score obtained with Solexa_Hypo is due to the fact that this library is created from C57BL/6 mice, the same mice strain that has been used to sequence the mouse genome while the Sanger_Hypo library come from fvb mice. It is probably partly valid. However, Sandberg et al [17] have estimated at 1% the number of tags that are different between 2 mouse strains. Furthermore, we estimated the percent of tags that match with one mismatch on the C57BL6/J genome to be of 4.05% and 2.29% for the Sanger_Hypo and the Solexa_Hypo libraries respectively. This is a very low percentage, thereby confirming the very low probability that an erroneous tag matches to the genome. This percentage is only slightly higher for Sanger_Hypo indicating that the better score obtained with Solexa_Hypo is not only due to strain differences.

**Figure 3:**
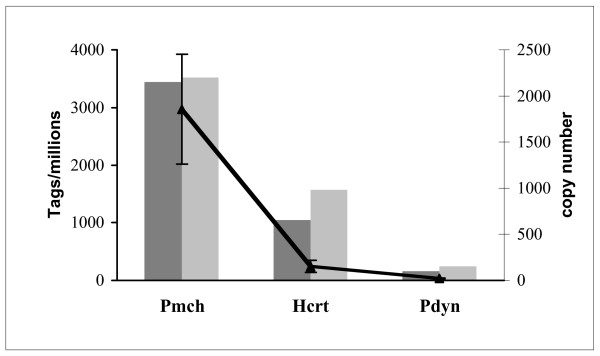
**Expression level for 3 well-known genes of the hypothalamus, using three different techniques**. The level of expression of three genes (pro-melanin concentrating hormone (Pmch), preprohypocretin (Hcrt) and prodynorphin (Pdyn)) known to be expressed in the hypothalamus is evaluated as their number of occurrence from the Sanger_Hypo library (darkest bars) and the Solexa_Hypo library (lighest bars) as tags per million (left axis). The level of expression of these 3 genes is also evaluated from 6 independent hypothalamic samples by qPCR (right axis). The mean and standard deviation are reported in copies of transcripts (right axis).

To validate the procedure of tissue collection, we looked at genes known to be absent in the hypothalamus but expressed in adjacent brain area such as the thalamus or the midbrain. Location was verified using the Allen Brain atlas of gene expression in mouse [18]. We selected the NMDA NR2C, the chloride channel calcium activated 2 and the sodium voltage gated type V alpha. None of them were found in the Sanger_Hypo or the Solexa_Hypo libraries. As libraries were constructed from two different hypothalamic samples, two different strains of mice and an amplification step was added for the built-up of the Solexa_Hypo library (Table 1), a direct comparison of the level of expression of selected genes is meaningless. We nevertheless checked for the expression level of 3 well-known genes of the hypothalamus, the pro-melanin concentrating hormone (Pmch), preprohypocretin (Hcrt) and prodynorphin (Pdyn) and found that in both libraries (Sanger_Hypo and Solexa_Hypo) and with q-PCR, the level of expression of Pmch is remarkably higher than Hcrt that is greatly elevated compare to Pdyn (Figure 3). This demonstrates the overall agreement between those three techniques.

**Figure 4:**
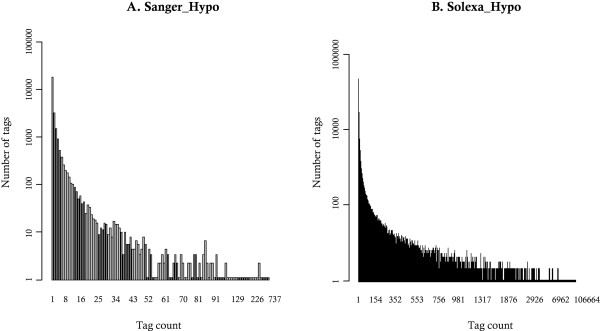
**Repartition of the number of tags according to their occurrence number in the Sanger_Hypo and Solexa_Hypo libraries**. A: Barplot for the Sanger_Hypo library, a mouse hypothalamic LongSAGE library sequenced by the Sanger method containing 68,023 total tags. B: Barplot for the Solexa_Hypo library, a mouse hypothalamic LongSAGE library sequenced by the Solexa technique containing 11,017,712 total tags. Please note that the Barplot representation displays only the observed values (if no tag is observed for a given count, the null Y value is not reported).

Since both libraries have been generated from the same tissue, we were however able to compared the distribution of tag occurrence between the two libraries. This distribution is highly similar between Sanger_Hypo and Solexa_Hypo libraries (Figure 4). A high proportion of tags is present in only one copy in the Sanger_Hypo library (68%) while they represent only 32% of the tags of the Solexa_Hypo library (Table 1), confirming that the depth of sequencing of the Sanger_Hypo library is not sufficient. It has been previously reported [19] that the distribution of large scale expression data is skewed by many low abundance transcripts. This has lead to the conclusion that all genes are expressed in all cells [19], although at a very low abundance, a process also known as «illegitimate transcription» [20]. Furthermore, we are dealing with populations of cells, harboring stochasticity detectable at the single-cell transcriptome level [21]. Finally, one also knows that part of the tags with a count of one is simply sequencing errors. Taken together, all those reasons are probably combined to produce the "classical" transcriptome profile displayed on Figure 4, which never shows its finite nature.

When we consider all unique tags from the two libraries combined, 2.8% of these tags are found in both libraries, 96.3% are found only in the Solexa_Hypo library, and 0.9% are found only in the Sanger_Hypo library. As one would expect, the mean occurrence number of the 2.8% common tags is higher (51.4 tpm in Sanger_Hypo and 34.2 tpm in Solexa_Hypo library) than the mean occurrence number of all tags (43.5 tpm in Sanger_Hypo and 1.6 tpm in Solexa_Hypo) in both libraries. Furthermore, if we select the 100 most abundant unique tags from both libraries (See Additional file 1), the number of unique tags found in both libraries is greatly increased (46%). These data confirm that the depth of sequencing of the Sanger_Hypo library is not sufficient to sample the tags present in the initial hypothalamic sample. The 0.9% of tags found uniquely in the Sanger_Hypo library might be mostly due to mouse strain differences (this is concordant with the estimation of 1% of transcriptomic differences between mouse strains by Sandberg et al. [17]).

### Depth of sampling

**Figure 5:**
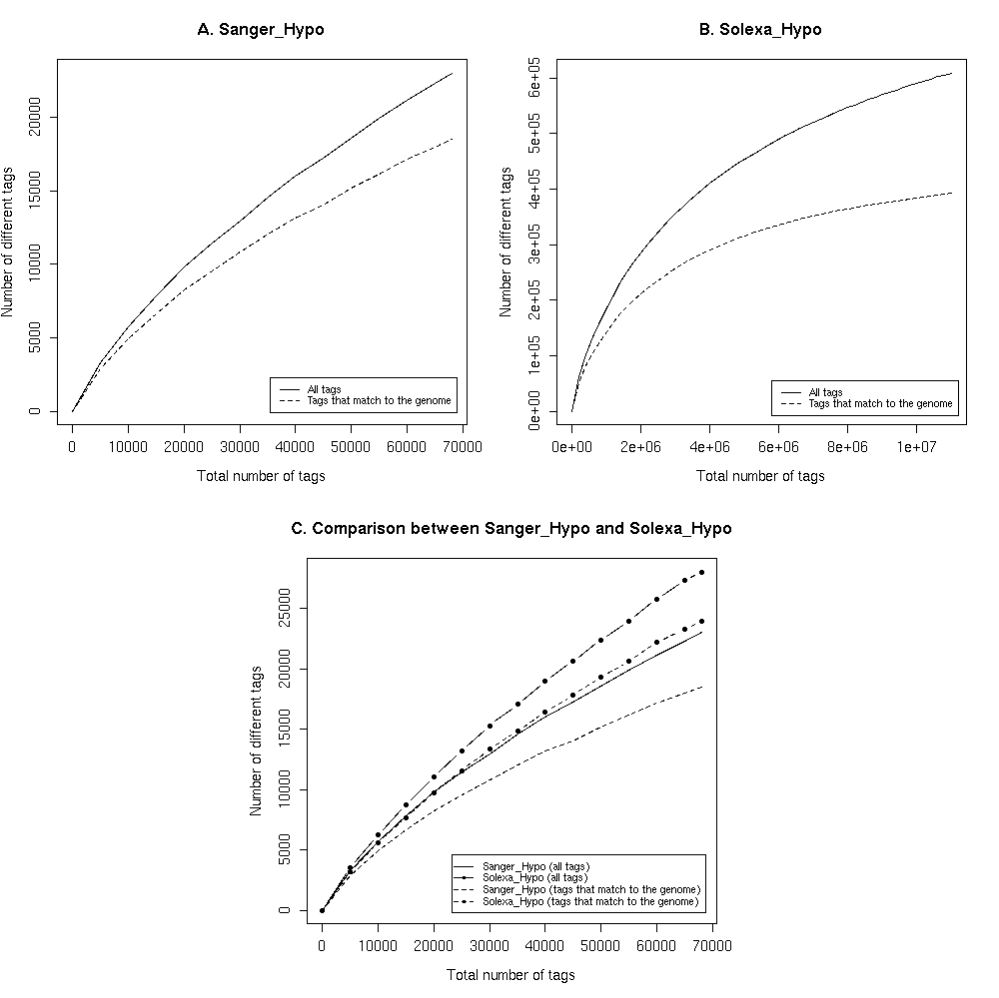
**Effect of the library size on the number of unique tags identified**. The three figures represent the number of unique tags identified as a function of the total number of tags in random libraries. These libraries were obtained by random sampling of X tags in the library considered (Sanger_Hypo or Solexa_Hypo), where X vary from 1 to the total number of tags in this library. In each of the obtained samples, we also calculated the number of unique tags that matches to the mouse genome (dotted lines). We considered that a tag matches to the genome when it has 100% identity over its whole length (21 bp). A: Figure for the Sanger_Hypo library. B: Figure for the Solexa_Hypo library. C: Figure comparing the number of unique tags identified as a function of the total number of tags between the Sanger_Hypo and the Solexa_Hypo library. The size of the random samples varies consequently from 1 to the size of the Sanger_Hypo library (the smallest of the two libraries).

To analyze the depth of transcriptome sampling in the Sanger_Hypo and Solexa_Hypo libraries, we studied the rate of increase of the number of unique tags identified as the size of the corresponding library increases (Figure 5). As shown in Figure 5A, this rate of increase is still high, even when the library size reached the total number of tags in the Sanger_Hypo library. This suggests that we are far from having distinguished each potential tag sequence of the initial hypothalamic sample. In contrary, the rate of increase of the number of unique tags identified decline drastically as we consider several millions of tags from the Solexa_Hypo library (Figure 5B).

Theoretically, this rate should equal zero if all unique tags of the initial hypothalamus sample had been sampled. However, it could be slightly greater because the addition of new tags could be due to the accumulation of different sequencing errors as the size of the library increases. We thus calculated the number of unique tags that matches to the genome in each of the random sampled libraries (dotted lines in Figure 5). As the probability that an erroneous tag matches to the genome is very low [3], this set of matching tags should contains almost only reliable tags. There is a smaller increase of the number of unique tags identified as the size of the library increases when we only consider matching tags than when we consider all tags (compare lines and dotted lines in the Figure 5). Moreover, the dotted line in the Figure 3A confirms that the size of the Sanger_Hypo library does not sample all unique tags from the initial hypothalamic sample. In contrary, it seems that we have almost sampled all different tags in the Solexa_Hypo library since the rate of discovery of new unique tags is severely dropping as the size increases: with a library size of one million, we were able to identify only one third of the unique tags (about 1.4 × 10^5 ^unique tags detected out of the total of 3.9 × 10^5 ^observed for the full library) whereas 80% of these tags were identified with a library of 5 millions of tags, and the vast majority (98%) in a 10 millions tags library (Figure 5B)."

### Complexity of the transcriptome

Increasing sequencing depth is only valuable if it gives access to a better image of the transcriptome complexity (i.e. the unique tags in the analyzed sample). We therefore compared the transcriptome complexity for the same random library sizes in the Sanger_Hypo and in the Solexa_Hypo SAGE libraries (Figure 5C) and found that LongSAGE combined either with Sanger or with Solexa sequencing gives access to similar transcriptome complexity. The number of unique tags identified in the Solexa_Hypo library is even slightly larger than the one in the Sanger_Hypo library (see the dotted lines on Figure 5C).

Hene et al. [11] showed that at a same sampling depth, a LongSAGE library of human T cells sequenced with the Sanger method contains much more unique tags than a MPSS library of T-cells (71,838 and 9,723 unique tags matching to the human genome for a sample size of 500,000 tags respectively). When we consider the same library size as Hene and colleagues, we sampled 118,075 unique tags matching to the mouse genome from the Solexa_Hypo library. Although a significant part of this difference may be accounted to the higher complexity of the hypothalamic sample used for the Sanger_Hypo and Solexa_Hypo libraries compared to the T cell sample used by Hene et al. [11], it does not explain it all. It rather advocates that LongSAGE – Solexa does not have the limitation of MPSS in its ability to explore the transcriptome complexity. Indeed, at a same sampling depth of 60,000 tags, considering only tags that match the genome, we found 21,133 unique tags in the Sanger_Hypo library and 25,604 unique tags in the Solexa_Hypo library (20% more), suggesting an ability of LongSAGE – Solexa to uncover more transcriptome complexity than LongSAGE – Sanger method.

## Conclusion

The present study shows that the Solexa sequencing technology is well adapted to the sequencing of LongSAGE tags, allowing to rapidly obtain a very deep LongSAGE library without the complexity limitation observed in MPSS libraries. The combination of LongSAGE and Solexa sequencing technology is therefore perfectly suited for deep transcriptome analysis.

## Methods

### Animals

Adult male mice (12–14 weeks of age), kept on a 12:12 light-dark cycle (light on at 7 am), were sacrificed by decapitation following the ethical committee's instructions (BH-2006-06) between 10 and 11 am. Brains were rapidly removed and frozen on dry ice.

Twenty-two fvb mice were used to create the I-Long SAGE hypothalamic library (Sanger_Hypo). Six C57BL/6 were used to make the LongSAGE-Solexa hypothalamic library (Solexa_Hypo). Six other C57BL/6 mice were used to perform qPCR controls.

### Collection of hypothalamic tissue

Brains were sliced in 400 μm-thick frontal sections at -12°C using a MICROM HM550 cryostat. The hypothalamus was collected bilaterally as 1 mm diameter punches centered on the perifornical nucleus from the caudal part of the paraventricular nucleus to the mammillary bodies (2 sections/animal) (Figure 2). Tissue samples were kept at -80°C until use.

### Total RNA extraction

Total RNA was extracted from a pool of hypothalamic tissue using the RNeasy mini kit (QIAGEN) following manufacturer's protocol. An average of 3.4 mg of hypothalamic tissue per mice was collected giving approximately 1 μg of total RNA per mg of tissue.

The quality and quantity of total RNA were assessed with the bioanalyzer 2100 (Agilent) and with optical density (Biophotometer, Eppendorf). The ribosomic RNA 28 s/18 s ratio was 1.76 and 1.98 for the Sanger_Hypo and Solexa_Hypo libraries respectively and RIN were over 8, indicating a suitable quality for the extracted RNA. The ratio of 260/280 on the biophotometer was over 1.8.

### RNA amplification

Since we are ultimately interested in using the longSAGE-solexa technology from small samples in upcoming studies, we added an amplification step to the protocol. Briefly, 100 ng of total RNA from hypothalamic samples of C57BL/6 were amplified using the SMART™ mRNA amplification kit (Clontech) to construct the Solexa_Hypo library following manufacturer's instructions.

The efficacy of the first and second strand cDNA synthesis was evaluated and quantified by looking whether the 5'end of a long sized gene is amplified with the same amount as the 3' end using the 1.2 kb GAPDH gene (3' end primers: 5'-AAGGTCATCCCAGAGCTGAA and 5'-TGTGAGGGAGATGCTCAGTG; 5' end primers: 5'-CGTCCCGTAGACAAAATGGT and 3'-GTGGTTCACACCCATCACAA). PCR amplifications were made using platinium^® ^Taq polymerase (Invitrogen) (PCR buffer Minus Mg 1×, dNTP 0,2 mM each, MgCl2 1,5 mM, primer 0,2 μM each, Taq 1 U, water qsp 50 μl; Primary denaturation at 94°C for 2 min, then 30 cycles of denaturation at 94°C for 30 sec, annealing at 60°C for 30 sec, extension at 72°C for 45 sec; expected sizes were 443 and 420 bp for 3' and 5' primers respectively). No difference in bands intensity was seen.

Amplification linearity was also evaluated using semi-quantitative PCR (Light Cycler^®^, Roche) by comparing the abundance of some hypothalamic genes before and after amplification calculated as the ratio between the level of expression of genes of interest normalized with GAPDH or cyclophillin before and after amplification. This ratio was comprised between 0.8 and 1.2 indicating that the amplification between genes was quite linear. The genes tested were prodynorphin F:TAGCTGAAGGAGAGACTGTC, R:CTGGGTTACTTGAATCCAGC; preprohypocretin F:CTAGAGCCACATCCCTGCTC, R:GGGAAGTTTGGATCAGGACA; NARP F:GCCTTTGTTGGAGAGCTCAG, R:AGAGGGCAGCTACAAGTCCA); Cyclophilin (F: CTGCACTGCCAAGACTGAATG and R: TTGCCATTCCTGGACCCAAA) and GAPDH (F: TCGTGGATCTGACGTGCCGCCTG, R: CACCACCCTGTTGCTGTAGCCGTAT).

### Quantitative RT-PCR

To evaluate the level of expression for 3 well-known genes expressed in the hypothalamus and compare it to the occurence of the corresponding tags of these genes in each library, we conducted a quantitative RT-PCR using SyBr green labeling (Light Cycler^®^, Roche). Reverse transcription was processed from 1 μg of total RNA extracted from C57BL/6 mouse hypothalami (n = 6) using the Superscript II reverse transcriptase (Invitrogen) following manufacturer's instructions. A PCR reaction was done using specific primers for pro-Melanin Concentrating Hormone (F:GTATGCTGGGAAGAGTCTAC, R:ACGTCAAGCATATCGCTTAC), preprohypocretin (see upper) and prodynorphin (see upper). Products of PCR were gel purified and the concentration in copy number was calculated for each gene. These samples were used to built-up a standard curve. Q-PCR was then done on the hypothalamic samples and on standards. By comparing values from hypothalamic samples to the standard curves, the level of expression of these three genes was evaluated in number of copies.

### I-Long SAGE

LongSAGE library construction was performed from 10 μg of total RNA using the I-LongSAGE™ kit (Invitrogen, Carlsbad, CA, USA) according to the manufacturer's protocol and as described in [22] (Figure 1). All control steps suggested in the kit were done using GAPDH PCR primers (F:5'-TTAGCACCCCTGGCCAAGG-3'; R:5'-CTTACTCCTTGGAGGCCATG-3') and platinium^® ^Taq polymerase (Invitrogen) (annealing of 55°C; 540 bp amplification product). The average insert size of concatemers was of 1119 bp, resulting in a mean number of 11.6 tags per concatemer. Sequencing was performed on 8448 clones by the Centre National de Séquençage (Genoscope d' Evry, France) by the SANGER method with an A3730 sequencing system. Among them, 6947 clones gave a proper sequencing results with 39324 ditags extracted. When eliminating repeated ditags, 36157 ditags were kept for subsequent analysis identifying a total of 68023 tags.

### LongSAGE Solexa

Five hundred nanograms of amplified RNA were processed to obtain the Solexa_Hypo library. Briefly, polyadenylated amplified RNA were fixed to oligo(dT) magnetic beads, first strand and second strand of cDNA were synthesized according to the first steps of the I-Long SAGE procedure used above (Invitrogen). The efficacy of the first and second strand cDNA synthesis was evaluated and quantified by PCR using GAPDH primers and platinium^® ^Taq polymerase (Invitrogen) as described above. The next steps were performed by GATC Inc. using their own adapters to be compatible with the Solexa high throughput sequencing technology. Similarly, their method is based on NlaIII and MmeI enzymatic digestions to isolate tags (Figure 1). Sequencing was performed using Solexa Illumina sequencing technology (GATC Inc).

### Bioinformatic analysis

For the Sanger_Hypo library, we used R and the Sagenhaft library [23] to extract ditags and tags from Phred concatemer sequence files. For the Solexa_Hypo library, we implemented a Perl script for tag extraction from Solexa sequencing files. In both libraries, we only considered tags with all bases having a probability of erroneous call (p_e_) of less than 1%. This correspond to a minimal phred score S_phred _= -10 log_10_(p_e_) = 20 and to a minimal Solexa sequencing score S_Solexa _= -10 log_10_(p_e_/(1-p_e_)) = 19.96. The percentage of tags with at least one base having a probability of erroneous call of less than 1% (ie Solexa score < 19.96) is of 69.59% (i.e. 11017712 tags out of 15831570 sequenced tags were of acceptable quality).

We matched tags on the mouse genome (Ensembl release 47, based on NCBI m37 assembly) by using the Megablast algorithm [24]. Only matches with 100% identity over the whole length of the tags (21 bp) were conserved, except for estimating the "one base mismatch" rate.

We used R to obtain random samples of tags from each library and to analyze corresponding results. All the graphics were done with R.

## Authors' contributions

LH, CF and NG built and CD and PW sequenced the Sanger_Hypo library. LH, CF and CSB built the Solexa_Hypo library. CK performed all bioinformatics analysis of the libraries. CP and OG provided guidance on this study. LH, CK, CP and OG participated in the writing of the manuscript. All authors read and approved the final manuscript.
